# The trade-off behaviours between virtual and physical activities during the first wave of the COVID-19 pandemic period

**DOI:** 10.1186/s12544-021-00473-7

**Published:** 2021-02-10

**Authors:** Elisa Bin, Claudia Andruetto, Yusak Susilo, Anna Pernestål

**Affiliations:** 1 Integrated Transport Research Lab, KTH Royal Institute of Technology, Stockholm, Sweden; 2 University of Natural Resources and Life Sciences (BOKU), Vienna, Austria

**Keywords:** COVID-19, Behavioural change, Internet usage, Digital infrastructure, Environmental and social sustainability, Virtual activity

## Abstract

**Introduction:**

The first wave of COVID-19 pandemic period has drastically changed people’s lives all over the world. To cope with the disruption, digital solutions have become more popular. However, the ability to adopt digitalised alternatives is different across socio-economic and socio-demographic groups.

**Objective:**

This study investigates how individuals have changed their activity-travel patterns and internet usage during the first wave of the COVID-19 pandemic period, and which of these changes may be kept.

**Methods:**

An empirical data collection was deployed through online forms. 781 responses from different countries (Italy, Sweden, India and others) have been collected, and a series of multivariate analyses was carried out. Two linear regression models are presented, related to the change of travel activities and internet usage, before and during the pandemic period. Furthermore, a binary regression model is used to examine the likelihood of the respondents to adopt and keep their behaviours beyond the pandemic period.

**Results:**

The results show that the possibility to change the behaviour matter. External restrictions and personal characteristics are the driving factors of the reduction in ones' daily trips. However, the estimation results do not show a strong correlation between the countries' restriction policy and the respondents' likelihood to adopt the new and online-based behaviours for any of the activities after the restriction period.

**Conclusion:**

The acceptance and long-term adoption of the online alternatives for activities are correlated with the respondents' personality and socio-demographic group, highlighting the importance of promoting alternatives as a part of longer-term behavioural and lifestyle changes.

## Introduction

During the first wave of the COVID-19 pandemic, the regular daily activity and worldwide economy have been disturbed by lockdowns of societies and cities to reduce the spread of the virus [5, 8, 20]. To cope with such a disruption, digital solutions have become an alternative for many people to fulfil their obligatory (e.g. working, studying) and non-obligatory (e.g. leisure, culture and sports) needs [2]. The ability of people to adopt and adapt to the digitalised alternatives is, however, different across socio-economic and socio-demographic groups as well as across types of occupations and branches. For some occupations, the changes from physical to virtual alternatives are almost straight forward, whilst for others, they are impossible, stressful, and they significantly deprive people’s livelihood and well-being, in particular for the disadvantaged groups [11, 33].

Whilst there have been dozens of studies which have accumulated and evaluated the transport, health, and well-being impacts of COVID-19 pandemic to different regions [41], sectors [15], and cities around the world [4, 13, 43], there is lack of knowledge on how people re-scheduled their activities and travel under different restriction conditions. In order to evaluate the impacts of the pandemic and the containment measures in the short and long term, it is important to understand the trade-off behaviours between virtual and physical activities during this disrupted period across different socio-demographic groups. Furthermore, it is important to understand the potential of digitalised solutions in creating more resilient transport systems, across socio-demographic groups, given different restriction characteristics.

The objective of this study is to contribute to this knowledge by filling this gap. In this study, we aim to investigate how individuals from different socio-demographic groups have changed their daily activity-travel patterns during the first wave of the COVID-19 pandemic and if these short-term behavioural changes could lead to long-term behavioural changes after the pandemic period. Moreover, we discuss the impact of the changes on social and environmental sustainability.

By using 781 responses to an online questionnaire, mainly from Italy, Sweden and India, this study investigates how individuals have changed their activity-travel patterns, during the first wave of the COVID-19 pandemic; the roles of the digitalised solutions in replacing physical activities, and behavioural changes that may be kept after the pandemic period over.

The next section provides a literature review on telecommuting/remote working, online shopping, and previous studies on the impacts of the pandemic on travel behaviour, followed by a section that describes the data collection. The profiles of the respondents and a descriptive analysis of the collected data are provided. Further, multivariate analysis is used to explore the short- and long-term changes in travel patterns and how these relate to the respondents’ changes in internet usage. The work then is concluded by the discussion and conclusion sections.

## Literature review

### Telecommuting and online shopping as digital alternatives for travel

Digitalised alternatives to travel and activity participation, such as remote working (telecommuting) and online shopping are not new. Remote working (or telecommuting), for example, has been identified by the transport researchers and planners as one important Travel Demand Management (TDM) strategy for reducing road congestion and vehicle emissions, and thus saving energy and improving air quality, since almost five decades ago [35]. Potential beneficial transportation impacts of telecommuting include reduced highway traffic congestion, reduced emission of pollutants, and energy and petroleum consumption savings. The actual amount and impact of telecommuting in any particular region, however, depends on travel demand measures in place and other aspects such as local condition and local transportation and land use environment [1]. Examples of recent reviews of different telecommuting settings and impact analyses can be found in Circella & Mokhtarian [9], Gössling, [17], Hook et al. [19], Moeckel [30], O’Keefe et al. [36].

Telecommuting has been rapidly developing as an acceptable way of working in various countries, in particular for certain occupations [18, 23, 31, 37, 42]. Statistics from the European Union (EU) in 2019 show an average of 5.3% of employed persons aged 15 to 64 usually work from home. This figure was highest in the Netherlands and Finland (14.1%), followed by Luxembourg (11.6%), and lowest in Bulgaria (0.5%) and Romania (0.8%) [14]. The percentage of employed persons in the EU who sometimes work from home has increased steadily over the years, from 7.7% in 2008 to 10.8% in 2019. Overall, in the EU, more self-employed persons usually worked from home (18.1%) than employees (9.6%). This was true in all Member States [14].

At the same time, with the rise of connectivity and telecommunication technologies, various online activities, including online shopping, have developed over the last 3–4 decades. A substantial number of efforts have been made to conceptualize the interactions between our transport systems and the telecommunication-based activities. Mokhtarian [32] and Salomon [42] used four definitions to describe possible relationships between telecommunications and travel. i) *Substitution* assumes that some demand for travel is replaced by a telecommunication alternative. ii) *Modification*, in contrast, anticipates the introduction of telecommunications technology to increase the use of transportation systems (for example, trip characteristics are modified by using telecommunication technologies to shift commute timing, location, linking and trip chaining without affecting the total amount of travel). iii) *Complementarity* refers to the situation where both transportation and telecommunications systems enhance the efficiency of each other. iv) *Neutrality*, means that telecommunication has no foreseeable effect on trip making. In analysing the impacts of telecommunication technologies, Mokhtarian [32] and Salomon [42] classified them into three period classifications: *short-term direct*, *short-term indirect*, and *long-term*. Mokhtarian, [32] considers short-term direct impacts as the possible substitution or stimulation of travel due to telecommunications. Short-term indirect impacts arise if time-savings from the replacement of travel by telecommunication are used to generate other trips. Long-term impacts are associated with the changes of land use patterns facilitated by telecommunications. Theoretical studies examining the impacts on residential location and urban development [27, 47] indicate that telework may also contribute to increased urban decentralization, and even generate more transport. Since then, the research on the interactions between transport and telecommunication technologies has become an established domain by itself, and a good overview of it can be seen at Liu et al. [26], Shen et al. [44], Shi et al. [45], Smidfelt Rosqvist & Winslott Hiselius [46], Suel & Polak [48].

### The roles of telecommuting, online shopping and other digital alternatives, during COVID-19 outbreak period

As discussed above, whilst telecommuting and online shopping have been introduced and operationalised for decades, like many other digitalised activities (e.g. online entertainment, distance learning), the adoption of such technologies has been relatively limited to only specific segments of the population. This, however, changed significantly when the digital alternatives were adopted as a strategy to contain the spread of COVID-19 pandemic.

With the continuous and fast COVID-19 virus spread at the beginning of the year 2020, between the end of February 2020 to the mid of March 2020 many countries around the world started to implement extreme restriction measurements and lockdowns of cities and societies [12]. The restrictions included the closing of activity places (e.g. schools, universities, non-essential work offices, non-essential stores, restaurants), parks, countries, and regions’ borders. In some regions, it was even forbidden to travel outside the municipality of residence (except for certified working reasons or severe health conditions). Countries are different between each other in terms of cultural, social, political and economical aspects; therefore the lockdown strategies are diverse. An overview of the major countries reached by our survey and the list of the restrictions in place can be found in Appendix A Differences in cultural, political and contemporary social contexts in the major countries reached by our survey and Appendix B Restrictive measures in place when the data have been collected (20th April – 18th May 2020) in the major countries reached by our survey.

The early impact of these COVID-19 containment measures on travel patterns and mobility have been studied, mostly based on secondary data, e.g. mobile phone and smart card data [10, 16, 21, 22, 24, 25, 34, 38, 39, 52]. Providing a full review and analysis of these results is beyond the scope of this paper. Instead, we provide a brief overview of the main findings. In Sweden, Dahlberg et al. [10] report a 64% increase of population in residential areas during working hours, a 33% average decrease of daytime presence in industrial and commercial areas, and a decrease of max trip length by 38%. In comparison, in the US 10.7–27.1% spent the time during workhours at home [21]*.* In Italy, Pepe et al. [38] report a reduction by 50% of the total number of trips between provinces, and an average reduction of the radius of gyration by 50%. By analysing public transport and bike/walk data from 41 cities by using the application city mapper, Malik et al. [28] show a reduction of the mobility of 3.5% per day during March, with an additional average reduction of 23% in places where social distancing measures were applied. Furthermore, Malik et al. [28] show that social distancing measures reduce mobility, but that there is no difference if the measures are hard or medium.

On wider impact analysis, Sabat et al. [40] carried out a survey to understand the public sentiment towards the measures used for COVID-19 containment in seven European countries. They report that in general people do trust their governments. They also report higher worries and higher support for harder measures in the southern parts of Europe than in the northern parts. Furthermore, different personality traits [7] and political orientation [22] are reported to have an impact on level of mobility reduction. Chan et al. [7] show that females are more likely to stay at home. Regarding differences in mobility reductions related to income levels, the results diverge. There are studies showing that lower income levels lead to less mobility reduction [53], while other studies show that there are no major differences between regions with different socio-economic levels [10, 21].

The literature review above shows that the impacts of how the measurements that were imposed to control the spread of the COVID-19 virus have widely affected not only people’s daily activity-travel patterns, but also the livelihood and social interactions of our society. Within a matter of weeks, movements and activity participations have been significantly reduced and people have been forced to resort to online alternatives. Whilst for some occupations and population segments the changes from physical to virtual alternative(s) are almost straight forward, for others they are impossible and stressful [33]. According to Harvard Business Review, countries like Sweden, which have robust digital platforms in the society, were significantly better prepared to the work from distance compared to Italy and India. According to this study Sweden was the best positioned country in Europe, to do socially distant work in terms of robustness of the key digital platforms and use of cash free payment methods. On the other hand, Italy positions poorly among the European countries, with digital platforms that are not sufficiently resilient or robust. India was one of the countries least prepared to manage remote work when the lockdown was imposed [6].

There are not only differences in the technological preparedness for telecommuting during the COVID-19 pandemic, but also differences in cultural preparedness. In Sweden, working remotely was an option largely available in most working places even before the pandemic period started [14]. On the other hand, in Italy it was very rare before the pandemic, and when the lockdown period started a new regulatory framework was implemented in May 2020 [29], in order to guarantee this option to workers with children under 14 years old. This hesitation towards remote working is correlated to a resistance from the managers to allow employees to work independently without potential constant supervision, as it would be in a regular office space [3].

## Data collection

To address the research objective of the study, an empirical data collection with a focus on behavioural changes before and during the first wave of the pandemic, was deployed through online forms. The survey was released on 20th April 2020, and the analysis provided in this paper is based on responses collected up to 18th May 2020. To reach a wider audience, the survey was made available in multiple languages (English, Italian, Swedish). The English language was chosen to reach as many as possible around the world; the Italian language was chosen because Italy was one of the first European countries to be heavily affected by the COVID-19 virus outbreak; and the Swedish language because the research work has primarily been in Sweden and because the policy adopted by the Swedish government is different compared to most of the other European countries. The survey was circulated through different groups on social media platforms as well as through the research centre network. The respondents were recruited via a convenience sampling method, as during the lockdown period, there was no time or choice to resort to a more selective method. The focus was on getting fast responses to capture the early changes during the first breakout of the pandemic. Since the survey was available in a few languages and due to the recruiting strategy employed, the respondents cover limited areas in Italy, Sweden and India.

The survey is divided into six sections, targeting respectively: i) change in travelling behaviour to perform daily activities (commuting, grocery shopping, non-grocery shopping, order take away food, eat out, visit friends and family, go out for entertainment/hobbies, physical activities); ii) change in internet usage (entertainment, personal call, work or study, work or study meetings); iii) change in online shopping behaviour (grocery and non-grocery); iv) perceived safety in performing daily activities (travelling by public transport, travelling by car, visiting stores, being at the workplace or school, going to restaurants, pubs and cafés, going to the gym, spending time outside, receiving home deliveries); v) intention of keeping the new habits (travel and commuting, grocery and non-grocery shopping, work or study, handle meetings at work or school, free time, physical activities) after the pandemic; and vi) personal information. The content of the survey is available in Appendix C- Survey structure.

## Results

Within 1 month of survey deployment, we received 781 valid responses of whom 53.6% of the respondents live in Italy, 28.9% in Sweden, 8.8% in India, and 8.7% in other countries.^1^ 51.4% are females, 67.1% are employed, and 74% are highly educated people. An overview of the demographic distribution in different countries is shown in Table 1. It is important to note that, whilst distribution across gender, education, and employment status in the total population is fairly reasonable, the distribution across those indicators in Sweden, Italy and India are not nationally representative. These differences are treated with care in the analysis. Moreover, in a later part of this paper, a multivariate approach is used to control for the impacts of each of these variables on the reported behaviours.

**Table 1:** Demographic distribution

		Total	Sweden	Italy	India	Other
Residence	–	**100%**	28.9%	53.6%	8.8%	8.7%
Gender	Female	**51.6%**	42.9%	60.6%	29.0%	47.8%
	Male	**48.1%**	57.1%	39.4%	71.0%	49.3%
	Other	**0.3%**	0.0%	0.0%	0.0%	3.0%
Level of education	Less than high school	**2.4%**	0.4%	4.3%	0.0%	0.0%
	High school graduate, diploma or equivalent	**20.4%**	12.4%	27.9%	11.6%	9.0%
	Trade/technical/vocal training	**3.2%**	3.1%	4.3%	0.0%	0.0%
	Bachelor’s degree	**24.3%**	20.8%	21.0%	53.6%	26.9%
	Master’s degree	**38.9%**	40.7%	38.2%	24.6%	52.2%
	Professional degree	**2.9%**	4.4%	1.2%	7.2%	4.5%
	Doctorate degree	**7.8%**	18.1%	3.1%	2.9%	7.5%
Employment	Employee	**54.4%**	61.5%	51.8%	53.6%	47.8%
	Self-employed	**10.8%**	7.1%	10.5%	24.6%	10.4%
	Housewife/houseman	**1.3%**	0.0%	1.0%	4.3%	4.5%
	Student	**20.6%**	21.2%	20.3%	11.6%	29.9%
	Part time worker	**1.9%**	2.2%	2.1%	0.0%	1.5%
	Volunteering	**0.4%**	0.0%	0.7%	0.0%	0.0%
	Military	**0.0%**	0.0%	0.0%	0.0%	0.0%
	Retired	**7.7%**	6.6%	9.8%	4.3%	1.5%
	Unemployed	**2.9%**	1.3%	3.8%	1.4%	4.5%

Overview of the demographic distribution, with focus on the countries with more respondents (Sweden, Italy and India)

### Changes in habits

**Fig. 1:**
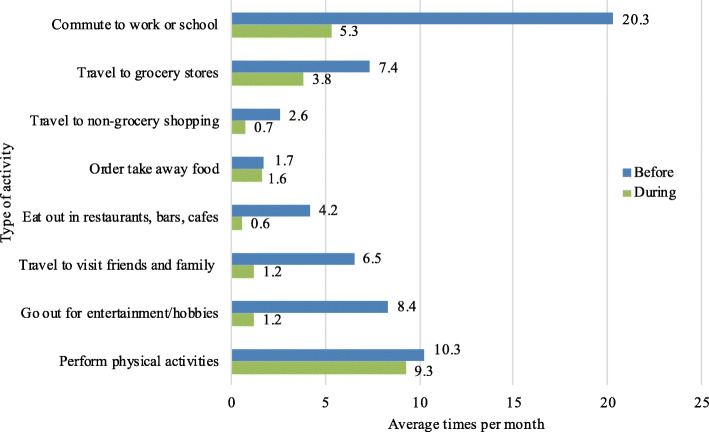
Frequency of different activities measured in times per month

Figure 1 shows how the frequency of travel for different activities has changed between before and during the COVID-19 virus outbreak. “Commute to work or school” is the activity that has been influenced the most, from an average of 20 trips per month to an average of 5 trips per month. However, the reduction of the number of trips for different activities differs significantly across countries. For commuting trips, it was reported a 71% decrease among Indian respondents, 69% in Italy, 80% in Sweden and 87% in the other countries. Whilst for the free time activity related trips, the Italian and Indian respondents reported a larger reduction in the number of trips, compared to their Swedish counterparts.

**Fig. 2:**
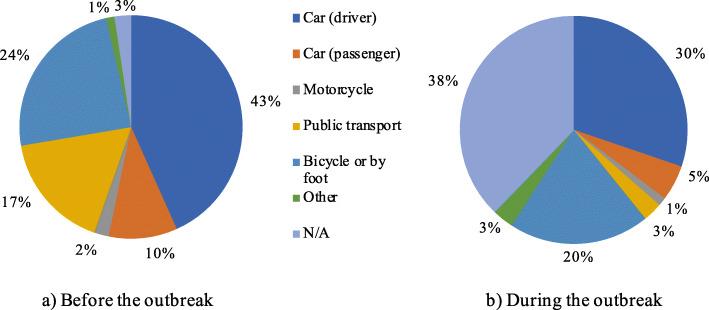
Mode share before and during the COVID-19 virus outbreak

The change of the most commonly used travel mode before and during the outbreak is shown in Fig. 2. Here, the mode share is calculated as the count of all the times the respondents selected a particular type of transport as travel mean for the given activity. The response “N/A” means that the respondent does not travel for a certain activity. The large increase in “N/A” during the outbreak in Fig. 2 represents the proportion of people who stopped travelling for some activities during the COVID-19 period. Figure 2 also shows a large decrease in public transport usage during the pandemic period.

**Fig. 3:**
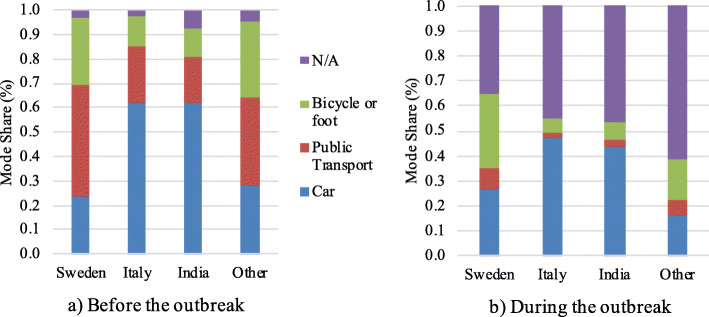
Mode share divided by country of residence for the commuting modes to work or school

Figure 3 shows the differences in mode share for commuting (to work or school) before and during the COVID-19 pandemic, for the different countries. In the figure, it can be seen that in Italy and India, where the car was the predominant before the crisis, it remained the most common mode in the cases where the trips are still performed, keeping into account that the responses “N/A” represent not travelling.

**Fig. 4:**
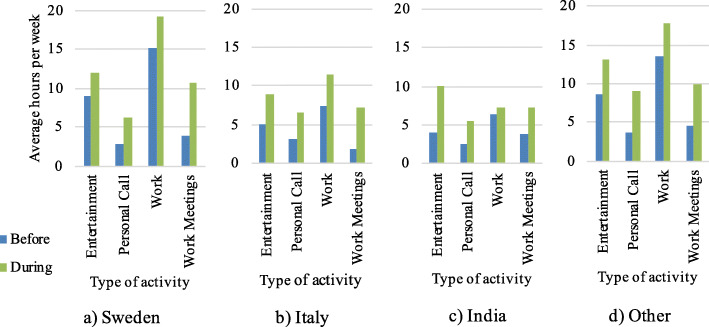
Average internet usage measured in hours per week, before and during the outbreak period

Figure 4 shows that there is a substantial increase in internet usage for all activities during the outbreak period, and in particular for meetings and calls related to work or study. The trend is consistent in all the countries. It should be noted that, compared to Italy and India, in Sweden the internet usage is higher on average both before and during the pandemic.

**Fig. 5:**
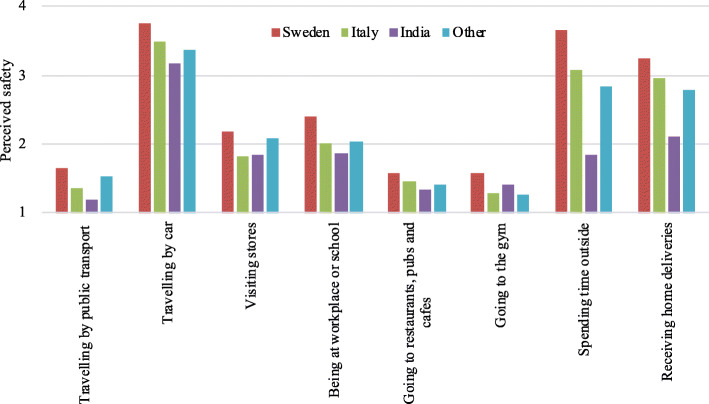
The respondents’ average perceived safety while engaging in the different activities from 1 (not safe at all) to 4 (very safe)

In Fig. 5, the average perceived safety of performing certain activities in different countries is shown. The perceived safety varies from 1 (not safe at all) to 4 (very safe). There is a trend that respondents living in Sweden perceived all activity participations safer than respondents living in the other countries, and in particular for the activities “spending time outside”, “receiving home deliveries” and “being at workplace or school”.

**Fig. 6:**
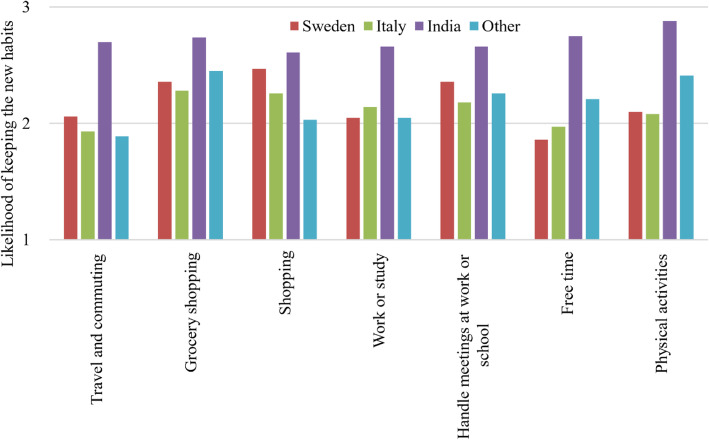
The respondents average reported likelihood of keeping their new habits in the different activities, from 1 (not likely) to 4 (very likely)

Figure 6 shows the respondents’ self-assessed likelihood to keep their new habits after the pandemic period, spanning from 1 (not likely) to 4 (very likely). Respondents that stated that they had not changed their behaviour are not included in this figure. In particular, the figure shows a higher claim among India respondents that they will keep their new habits after the pandemic period.

## Multivariate analysis results

To examine the impacts of individuals’ internal and external factors on their changed behaviour comprehensively whilst also controlling for the impacts of the biased sample and values of each influencing factor, a series of multivariate analyses was carried out. A series of regression models is presented in this section, related to the change of travel activities and internet usage, before and during the pandemic period. Furthermore, a binary regression model is used to examine the likelihood of the respondents to adopt and keep their behaviours beyond the pandemic period.

Three different tables were created: one for each trip purpose (Table 2), one for the change in internet usage (Table 3), and one for the likelihood of keeping the new behaviour after the pandemic (Table 4).

**Table 2:** Regression models for the decreased amount in monthly trips for different activities

	Variable ranges	Changes in commute trips		Changes in grocery trips		Changes in non-grocery trips		Changes in family and friends visit trips		Changes in eating out trips		Changes in hobby related trips		Changes in sport related trips	
Variable ranges		[−40, + 40]		[−40, + 40]		[−40, + 40]		[−40, + 40]		[−40, + 40]		[−40, + 40]		[−40, + 40]	
		B	t	B	t	B	t	B	t	B	t	B	t	B	t
(Constant)		−7.682	−2.24*	−3.208	−2.01*	− 2.909	− 2.54*	−6.979	− 3.14*	−4.944	− 2.78*	−9.186	−3.44*	−6.394	− 2.05*
Being a female	0, 1	0.014	0.02	−1.239	− 2.90*	− 0.151	− 0.51	− 0.571	− 1.02	− 0.464	− 1.06	− 0.175	− 0.25	1.326	1.61
Being a full-time worker	0, 1	−7.568	−5.27*	0.458	0.67	−0.328	− 0.68	− 0.087	− 0.10	− 2.982	−4.22*	− 0.946	− 0.85	0.834	0.63
Being a student	0, 1	− 10.216	−6.23*	0.203	0.26	0.182	0.33	0.264	0.25	−2.506	−3.04*	−1.981	− 1.56	2.832	1.87**
Have university education	0, 1	−1.259	−1.21	−0.563	− 1.13	0.476	1.35	−0.347	− 0.52	− 0.252	−0.48	0.200	0.25	0.337	0.35
Having children within household	0, 1	0.193	0.21	0.241	0.53	0.037	0.12	1.053	1.74**	1.167	2.50*	1.582	2.16*	−0.112	−0.13
Having adults within household	0, 1	−0.053	−0.06	0.358	0.78	0.159	0.50	0.228	0.38	−0.050	− 0.11	0.715	0.96	2.224	2.50*
Having elderly within household	0, 1	1.697	1.43	−1.755	−3.08*	−0.690	− 1.72**	− 0.477	− 0.63	0.270	0.46	− 1.085	− 1.18	− 0.399	− 0.36
Do not travel for the given activity during pandemic	0, 1	−4.984	− 3.44*	− 1.905	−2.48*	− 0.477	− 1.00	1.644	1.82**	− 0.085	− 0.11	−1.820	− 1.81**	− 2.057	− 2.08*
Living in Sweden	0, 1	1.146	0.67	1.031	1.26	1.353	2.29*	2.176	1.96*	1.628	1.87**	3.543	2.62*	2.469	1.54
Living in Italy	0, 1	2.472	1.52	1.091	1.36	1.896	3.41*	−2.025	−1.91**	1.106	1.32	−0.569	− 0.44	1.747	1.14
Living in India	0, 1	0.136	0.06	1.766	1.68**	0.778	1.08	1.587	1.16	2.161	2.01*	4.102	2.46*	−3.594	−1.80**
Used private car to carry out the given activity before pandemic	0, 1	−1.479	−1.04	1.569	2.43*	0.306	0.66	−0.515	− 0.58	1.002	1.53	0.497	0.56	0.152	0.14
Used public transport to carry out the given activity before pandemic	0, 1	−3.033	−2.32*	−0.886	− 0.79	− 0.749	− 1.53	− 0.806	− 0.79	0.865	1.23	0.846	0.80	−3.885	−2.18*
Used private car to carry out the given activity during pandemic	0, 1	2.278	1.36	− 0.829	−1.32	0.085	0.16	2.424	2.44*	1.761	2.06*	0.814	0.70	−3.338	−2.41*
Used public transport to carry out the given activity during pandemic	0, 1	−1.431	−0.57	2.663	1.25	1.418	1.54	2.105	1.17	0.984	0.68	−1.745	−0.82	−3.818	− 0.86
R		.415^a^		.225a		.201a		.287a		.273a		.299a		.243a	
R square		0.172		0.051		0.040		0.082		0.075		0.090		0.059	
Adjusted R square		0.156		0.032		0.022		0.064		0.057		0.072		0.040	
Std. Error of the estimate		11.865		5.725		4.021		7.613		5.926		9.277		11.140	
Number of samples		781		781		781		781		781		781		781	

The more negative t-coefficient, the larger relative decrease in number of trips, compared to their counterparts. One star (*) indicates 95% confidence level, two stars (**) indicates 90% confidence level. Variable ranges are indicated for each variable

**Table 3:** Regression models for the increased amount in daily internet usages for different activities

	Variable ranges	Change in use of internet for work meetings		Change in use of internet for entertainment		Change in use of internet for personal calls		Change in use of internet for work		Change in online shopping (groceries)		Change in online shopping (non-groceries)	
Variable ranges		[−32.5, + 32.5]		[− 32.5, + 32.5]		[− 32.5, + 32.5]		[− 32.5, + 32.5]		[− 100%, + 100%]		[− 100%, + 100%]	
		B	t	B	t	B	t	B	t	B	t	B	t
(Constant)		−0.688	−0.27	4.257	2.12*	4.152	2.15*	3.100	1.09	0.085	1.19	0.133	1.59
Being a female	0, 1	−1.608	−2.49*	0.762	1.55	−0.375	− 0.79	− 0.458	− 0.65	0.009	0.50	0.022	1.04
Being a full-time worker	0, 1	3.332	3.10*	1.123	1.42	1.265	1.65**	3.678	3.13*	−0.060	−2.10*	0.091	2.73*
Being a student	0, 1	4.625	3.84*	2.692	3.06*	3.425	4.02*	3.292	2.50*	−0.061	−1.92**	0.009	0.24
Have university education	0, 1	2.456	3.27*	1.036	1.82**	1.958	3.55*	0.256	0.31	0.039	1.89**	0.033	1.39
Having children within household	0, 1	0.600	0.88	−0.775	−1.49	0.345	0.69	0.323	0.43	−0.004	−0.19	0.010	0.46
Having adults within household	0, 1	−0.021	−0.03	0.091	0.17	−0.301	− 0.59	− 0.308	− 0.40	− 0.006	− 0.30	− 0.016	−0.71
Having elderly within household	0, 1	−1.012	− 1.16	− 0.852	− 1.29	− 0.804	− 1.26	−1.243	− 1.31	− 0.012	− 0.50	− 0.016	− 0.58
Living in Sweden	0, 1	1.797	1.45	−0.198	− 0.21	−1.225	− 1.33	− 0.243	−0.18	0.048	1.42	−0.043	− 1.07
Living in Italy	0, 1	1.664	1.40	0.381	0.42	−1.318	− 1.51	0.221	0.17	0.054	1.66**	−0.045	−1.19
Living in India	0, 1	−1.708	−1.10	1.592	1.31	−2.339	−2.00*	−3.434	−2.02*	0.071	1.67**	−0.386	−7.65*
Change in trips for the given activity	[−40, + 40]	−0.129	−5.03*	− 0.028	−2.35*	− 0.086	− 2.83*	−0.069	−2.45*	− 0.002	−1.35	−0.004	− 1.76**
Changes in eating out trips	[−40, + 40]	–	–	–	–	–	–	–	–	− 0.001	−0.85	–	–
Safety perceived in the workplace	0, 1	−0.170	−0.23	–	–	–	–	− 0.552	−0.69	–	–	–	–
Safety perceived outside	0, 1	–	–	− 1.996	− 3.06*	− 1.302	− 2.07*	–	–	–	–	–	–
Safety perceived in stores	0, 1	–	–	–	–	–	–	–	–	− 0.069	− 3.00*	− 0.076	− 2.81*
Safety perceived in receiving home-deliveries	0, 1	–	–	–	–	–	–	–	–	− 0.023	− 1.11	0.017	0.68
Safety perceived using public transport	0, 1	−2.039	−1.77**	− 0.456	−0.54	− 1.369	− 1.67**	−1.653	− 1.31	−0.020	− 0.59	0.006	0.15
Safety perceived using private car	0, 1	−0.477	−0.45	− 0.114	−0.14	− 0.496	−0.61	−1.401	− 1.20	0.032	1.07	−0.005	− 0.14
R		.336^a^		.244^a^		.263^a^		.220^a^		.198^a^		.388^a^	
R square		0.113		0.059		0.069		0.048		0.039		0.150	
Adjusted R square		0.097		0.042		0.052		0.031		0.019		0.134	
Std. Error of the estimate		8.712		6.62491		6.41037		9.52366		0.23686		0.27914	
Number of samples		781		781		781		781		781		781	

A larger t coefficient means a relatively larger increase in internet usage, compared to their counterparts. One star (*) indicates 95% confidence level, two stars (**) indicates 90% confidence level

**Table 4:** Binary logistic regression for a likelihood of keeping the behavioural changes after the pandemic period

	Variable ranges	Likelihood to keep the new work meetings habits		Likelihood to keep the new commuting habits		Likelihood to keep the new work habits		Likelihood to keep the new shopping habits (groceries)		Likelihood to keep the new shopping habits (non-groceries)		Likelihood to keep the new free-time habits	
Variables ranges		[1, 5]		[1, 5]		[1, 5]		[1, 5]		[1, 5]		[1, 5]	
		B	t	B	t	B	t	B	t	B	t	B	t
(Constant)		−2.457	−2.79*	−1.999	−2.41*	−4.575	−4.46*	−2.319	−2.79*	−5.101	−5.77*	−2.985	−3.01*
Being a female	0, 1	−0.326	−1.53	−0.433	− 2.13*	−0.343	−1.48	0.467	2.26*	0.085	0.42	0.342	1.44
Being a full-time worker	0, 1	0.291	0.73	−0.215	− 0.61	1.159	2.52*	0.186	0.53	0.406	1.15	−0.520	−1.32
Being a student	0, 1	−0.160	−0.37	− 0.284	−0.72	0.782	1.55	−0.142	−0.36	0.203	0.52	−0.290	−0.68
Have university education	0, 1	0.004	0.02	0.376	1.54	−0.253	−0.94	− 0.405	−1.72**	0.231	0.99	−0.531	−1.98*
Having children within household	0, 1	0.618	2.81*	−0.330	−1.57	−0.076	− 0.31	−0.084	− 0.39	0.288	1.40	0.413	1.64**
Having adults within household	0, 1	−0.433	−1.86**	0.279	1.29	−0.001	0.00	−0.065	−0.30	0.275	1.26	−0.131	−0.52
Having elderly within household	0, 1	0.216	0.73	−0.213	−0.76	− 0.002	−0.01	− 0.052	−0.19	0.250	0.92	0.402	1.30
Living in sweden	0, 1	−0.121	−0.30	0.450	1.15	−0.030	−0.06	− 0.868	−2.18*	1.201	2.85*	−1.408	−3.15*
Living in italy	0, 1	−0.520	−1.32	0.065	0.18	0.604	1.34	−0.457	−1.23	1.069	2.65*	−1.092	−2.62*
Living in india	0, 1	−0.011	−0.02	0.554	1.19	0.491	0.82	0.163	0.33	0.733	1.39	−0.189	− 0.34
Changes in trips for the given activity	[−40, + 40]	− 0.001	− 0.14	0.001	0.11	0.021	2.16*	−0.003	−0.15	0.021	0.89	0.009	1.56
Change in use of internet for work	[−32.5, 32.5]	0.012	1.00	0.003	0.29	−0.003	−0.20	–	–	–	–	–	–
Change in use of internet for work meetings	[−32.5, 32.5]	0.031	2.20*	−0.008	−0.66	0.024	1.61	–	–	–	–	–	–
Change in online shopping (groceries and non-groceries, respectively)	[−32.5, 32.5]	–	–	–	–	–	–	1.181	2.89*	0.998	2.93*	–	–
Change in use of internet for entertainment	[−32.5, 32.5]	–	–	–	–	–	–	–	–	–	–	0.030	1.57
Change in use of internet for personal calls	[−32.5, 32.5]	–	–	–	–	–	–	–	–	–	–	−0.032	−1.52
Likelihood to keep the new work meetings habits	[1, 5]	–	–	0.854	3.49*	2.879	12.15*	−0.486	−1.84**	0.731	3.01*	0.588	2.06*
Likelihood to keep the new commuting habits	[1, 5]	0.905	3.66*	–	–	0.791	3.15*	1.190	5.25*	0.654	2.98*	0.557	2.09*
Likelihood to keep the new work habits	[1, 5]	2.914	12.07*	0.715	2.89*	–	–	0.448	1.67**	0.264	1.06	0.903	3.10*
Likelihood to keep the new shopping habits (grocery)	[1, 5]	−0.462	−1.75**	1.191	5.27*	0.475	1.73**	–	–	2.051	9.92*	0.369	1.37
Likelihood to keep the new shopping habits (non- grocery)	[1, 5]	0.699	2.88*	0.633	2.86*	0.383	1.50	2.032	9.80*	–	–	0.697	2.65*
Likelihood to keep the new free-time habits	[1, 5]	0.523	1.84**	0.552	2.15*	0.938	3.23*	0.426	1.63	0.709	2.77*	–	–
Likelihood to keep the new sport habits	[1, 5]	0.070	0.26	0.105	0.43	0.202	0.71	0.596	2.42*	0.215	0.88	2.800	11.88*
Safety perceived in the workplace	0, 1	0.293	1.24	−0.225	−1.01	−0.427	−1.69**	–	–	–	–	–	–
Safety perceived in stores	0, 1	–	–	–	–	–	–	−0.020	−0.08	0.053	0.22	–	–
Safety perceived being outside	0, 1	–	–	–	–	–	–	–	–	–	–	0.658	2.04*
Safety perceived eating out	0, 1	–	–	–	–	–	–	–	–	–	–	0.184	0.42
Safety perceived using public transport	0, 1	0.036	0.09	0.091	0.25	0.278	0.69	−0.354	−0.89	−0.053	−0.15	− 0.517	−1.08
Safety perceived using private car	0, 1	0.230	0.65	−0.510	−1.55	0.898	2.09*	0.768	2.17*	−0.020	−0.06	0.062	0.15
−2 log likelihood		632.162^a^		693.947^a^		549.998^a^		675.508^a^		710.066^a^		532.951^a^	
Cox & snell r square		0.414		0.260		0.429		0.337		0.330		0.403	
Nagelkerke r square		0.560		0.373		0.598		0.468		0.452		0.577	
Number of samples		781		781		781		781		781		781	

We asked about the likelihood of keeping the new behaviour with multiple choice from 1 (not likely) to 4 very likely) and we grouped the responses into: “likely to keep the habit” (answered 3 or 4) and “not very likely to keep the habit” (answered 1 or 2). A larger t coefficient means a larger likelihood to keep the new habits, compared to their counterparts. One star (*) indicates 95% confidence level, two stars (**) indicates 90% confidence level

In Table 2,3 and 4, the variables in the columns are the dependent variables and the variables in the rows are the independent variables (or explanatory variables) in the model estimation. B is the estimated unstandardized regression coefficients and t is the t-test statistic that indicates the significance of each dependent variable in influencing the value of the given dependent variable. The independent/explanatory variables that significantly influence the dependent variable at 95% confidence level are indicated by a star (*), and the ones who are significant at 90% confidence level by two stars (**). The positive and negative significant coefficients in Table 2, 3 and 4 represent the relative changes. In Table 2, a negative coefficient means a larger decrease in travel, compared to their counterparts. A positive coefficient, on the other hand, means that the reduction in travel was relatively smaller. In Table 3, a larger coefficient indicates a larger relative increase in internet usage, compared to others. In Table 4, a larger coefficient means a larger likelihood to keep the new habit compared to their counterparts.

### Who had the largest reduction of trips and for which activity participation(s)?

The results in Table 2 show that the ones who stopped travelling for certain activities (whether it was imposed on them by external actors or by self-conscience) had a significant reduction in their commute trips, in particular full-time workers and students. The ones who used to commute with public transport before the virus outbreak, also have a larger reduction in their commute trip frequencies compared to their counterparts.

During the first wave pandemic period, females had a larger reduction of their grocery trip frequencies, compared to their male counterparts. It is presumably due to the changes in intra-households’ chores related trips during the pandemic period. Both full-time workers and students had larger reductions of their eating out trips, compared to others, whilst at the same time, students decreased their number of sport related trips less than their counterparts (at 90% confidence level).

Respondents with children (less than 18 years old) in their households have a relatively higher frequency of eating out, hobby related trips, and visits to friends and families (at 90% confidence level), compared to other households. Having adults within the household is correlated with a higher frequency of sport related trips, compared to the rest of the respondents, whilst having elderly (older than 65 years old) in the household is correlated with a lower frequency of grocery and non-grocery trips (at 90% confidence level).

Living in Sweden during the pandemic is associated with higher frequencies of non-grocery trips, family and friends’ visits, eating out trips (at 90% confidence level), and hobby related trips, compared to respondents from other countries. At the same time, living in India is correlated with a higher frequency of grocery shopping, eating out, and hobby related trips, and lower frequency of sport related trips. Compared to other countries, the Italian respondents report a smaller decrease in non-grocery shopping trips and a bigger reduction on family and friends related trips. In Italy, the number of non-grocery shopping trips decreased from 2 per month before the pandemic to 0.6 during the pandemic, while in Sweden non-grocery trips decreased from 3 to 1 per month. In terms of family visits, the average reported number of the Italians’ family and friend visits is 8.7 trips per month before the outbreak and 1.6 trips during the outbreak. For Sweden, the corresponding numbers are 3.6 and 0.9 trips per month.

Table 2 also shows that those who used the private car during the pandemic for family trips and eating out reduced these activities less. The ones who used public transport before the pandemic suffered a more significant reduction in commuting and sport related trips.

### Who had the largest increase of internet usage, and for what purpose?

Both men and women increased their internet usage for work meetings during the first wave of the pandemic period. However, in Table 3 it can be observed that women increased their internet usage less than men. In absolute values, the survey responses showed that women increased their weekly internet usage by 4.9 h during the pandemic, while men by 6.3 h.

Being a full-time, highly educated worker or a student is associated with a larger increase in internet usage during the pandemic period, than others. This includes online personal calls, work meetings and online groceries shopping. Internet usage for entertainment significantly increased among students during the pandemic period, and among highly educated (at 90% confidence level).

The results show that living in India during the pandemic period is associated with a lower increase in internet usage for work meetings, personal calls and non-grocery shopping, compared to other countries.

Table 3 shows that internet usage has substituted physical travelling and activities. Reductions of physical trips for certain purposes (e.g. when one has stopped commuting/physical grocery shopping/go for leisure related trips) lead to significant increases of similar activities online. At the same time, people that continued to commute did not increase their internet usage for work as much as their counterparts.

In Table 3 also shows that the perceived safety significantly influences internet usage for some activities but not for all. A higher perceived feeling of safety while being outside is correlated with relatively lower internet usage for personal calls and entertainment. Likewise, a higher perceived safety while being in stores is correlated with relatively lower internet usage for grocery and non-grocery shopping. On the other hand, how safe the respondent feels at work locations is not correlated to internet usage for work purposes.

### Who is likely to keep their changed behaviours beyond the pandemic period, and for what purpose?

The coefficients shown in.

Table 4 indicate that females consider themselves more likely to keep the new online shopping behaviours than their male counterparts. At the same time, females report that they are less likely to keep their new (online) work habits. This could be explained by the fact that females’ workplaces are more likely to be located closer to home, with a type of occupation which likely requires them to be in the office more often than their male counterparts [49, 50].

Being a full-time worker, as well as having a smaller reduction in the number of trips for commuting, is correlated with a higher likelihood of keeping the new work habits, compared with their counterparts. Whilst the university-educated respondents are more likely to keep their new free time habits, the respondents with children are more likely to keep their online working meeting habits.

Whilst Swedish respondents are more likely to keep their new non-grocery shopping habits, they are not likely to keep their new grocery and new free-time habits. Likewise, in Italy, the respondents are likely to keep their new non-grocery habits, but not their free-time habits.

## Discussion

### How perceived safety and behavioural change differ across respondents from countries with different restriction levels

In the study, respondents living in Sweden were found to be less worried than respondents living in other countries, whilst respondents living in Italy were found to be more worried. This result is in line with the findings by Sabat et al. [40], who report that people in northern Europe tend to be less worried compared to people living in southern Europe. The estimation results in Table 2 show that respondents from Sweden changed their behaviour for leisure activities (e.g. visit friends and family, eating out, travel to non-grocery stores and for hobbies) less than their counterparts in other countries. This result is in line with the restriction policies in place at the time of the survey, and also consistent with the trends published by Google Community Mobility Report [15]. The bigger changes in habits in Italy and India, however, are also presumably due to the strict lockdown imposed on the population at the time when we collected the data, which does not allow our respondents to do otherwise.

The results show that opportunity and possibility to change the behaviour matter. The ones who stopped travelling for certain activities (whether it was imposed on them by external actors or by self-conscience) are the ones who consistently had a significant reduction in their trips.

### Impact on social sustainability

Tirachini & Cats [51] describes concerns about future public transport ridership in relation to the way the public perceives it. In particular, seeing public transport as unhealthy and dangerous as a consequence of the pandemic might have long term implications on public transport and increase social discrepancies. However, our results indicate that one’s concern of using public transport is not the main variable that significantly influences one avoiding coming back to the old behaviours (travel and doing activities physically). In fact, from our analysis, that particular variable was found insignificant in influencing whether one would adopt the new (online) behaviours, after the pandemic period.

One important finding shown in Table 4 is the strong correlation between one’s intention of keeping one particular new (online) habit with one’s intention to keep other new (online) habits. This indicates that acceptance and long-term adoption of technological alternatives (in this case internet) is not necessarily tied to a particular purpose, but more to the personality and socio-demographic group of the given person. In turn, this highlights the importance of policy design and infrastructure development, which are not only suited for one particular activity-related purpose (e.g. work related), but promote the technological alternatives as a potential solution for long-term habits and lifestyle changes. This is one of the basic key factors for delivering digitalised infrastructure to create a more resilience transport and economic system in the future.

### Impact on environmental sustainability

During the pandemic period, there has been an increase in online shopping for grocery and non-grocery items, and people reported that they are likely to keep the behaviour after the pandemic (Fig. 6). These changes in shopping behaviour will lead to consequent changes for the logistics and the retails sectors. However, it is not obvious whether such changes contribute to sustainability or not as the number of people trips for shopping are likely to be reduced; they are replaced by freight transports. Furthermore, research shows that an increase in online shopping might induce other rebound effects (e.g. trips for other purposes, impact in public transport usage) [45].

Moreover, we can see an increase in internet usage and a substantial decrease in the number of trips. In Italy and India, where the mode share before the crisis was dominated by private cars, the reduced number of trips is likely to have a significant impact on emissions, and subsequently on the environment. The estimation results in Table 4 do not show any strong indication of the influence of the country of residence (and the corresponding restriction policy) on one’s likelihood to adopt the (new/online based) behaviours for all the activities after the restriction period. Whilst Swedish and Italian respondents are more likely to keep their new non-grocery shopping habits, the Swedish respondents are not likely to keep their new grocery and new free-time habits, whilst the Italian respondents are not likely to keep their new free-time habits.

### Caveats - comments on population reached in different countries

It is important to treat our results with caution, as our data is not representative of the given countries’ socio-demographic distributions. Among our sample population, data from Italy are more diverse in terms of socio-demographic groups, whilst our Swedish respondents are mainly highly educated people. For India, the number of respondents is too small to represent the actual population of the country. Although in general the gender distribution of the sample seems evenly spread between female and male respondents (51.6% female), 60.6% of the Italian respondents is female and 71.0% of Indian respondents is male. Therefore, the results of this study should be interpreted as indicative. Moreover, for this analysis, we are considering the data collection period (20th April – 18th May 2020) as homogeneous, without further distinguishing between sub-periods with different policies in place. This could have had an effect on the reported perceived safety of our respondents. Appendix B - Restrictive measures in place when the data have been collected (20th April – 18th May 2020) in the major countries reached by our surveydescribes the policies in place at the time of the study. We also highlight that most of our responses come from Italy and Sweden. In Italy, most of our data come from the north of the country, where there were more restrictive measures throughout the considered period and in Sweden, the lockdown policies never changed during the analysed period.

### Future work

There are several important topics that need to be learned on how peoples’ behaviour changed during the lockdowns and containment measures. Our future work includes a more detailed investigation of the roles of online shopping behaviours and its logistics companies. How the behaviours, values and technological acceptances changed overtime during the pandemic period is also an important topic to be investigated. We also see a need to explore further the long-term implications on social and environmental sustainability.

## Conclusion

By analysing 781 responses to an online survey, this study has investigated how individuals changed their travel patterns and use of online activities during the first wave of the COVID-19 pandemic during spring 2020. Descriptive analysis and regression models have been used to explore, among the respondents, who have changed their travel patterns, who have changed their internet usage, and who are intending to keep their habits after the pandemic period is over. Due to the nature of our responses, we also got the opportunity to explore the behavioural differences between our respondents who reside in Italy, Sweden, and India.

The four main take-aways from the study are listed below:

1. The ones who stopped travelling for one activity also reduced their travel for other activities. The results show that the ones with children in the household had smaller reductions in travel for eating out, hobbies, and visits to friends and family.
2. Online activities have replaced travelling to some extent. In particular, full-time workers, highly educated people, and students increased their internet usage more than their counterparts, while respondents in India reported less increase in internet usage.
3. Full-time workers and respondents with children in the household are more likely to keep their new online working habits.
4. Changes in behaviour were more considerable for respondents in Italy and India, while respondents in Italy and Sweden report that they are more likely to keep at least part of their new online behaviours.

To summarise, the results show that acceptance and long-term adoption of online alternatives tie to the personality and socio-demographic group of the given person, which highlights the importance of promoting alternatives as a part of more extended behavioural and lifestyle changes.

The impacts of the COVID-19 pandemic on social and environmental sustainability are still difficult to judge. In the short-term, the reduction of car travel is likely to contribute to fewer emissions, while the long-term effects still need to be further explored. The output of this study has provided insights on the opportunities for and the role of digitalisation (e.g. online services) in creating future sustainable and resilient transport systems.

## Data Availability

The datasets collected and analysed during the current study are not yet publicly available but are available from the corresponding author on upon request.

## Appendix A

### Differences in cultural, political and contemporary social contexts in the major countries reached by our survey

The data analysed in this work come from different countries, in particular, the majority of our respondents are from Italy, Sweden and India. These three countries are very different in terms of cultural, economic and social context. In this Appendix, we collected data regarding these differences.^2^

India is the most populated and dense between the three, followed by Italy and finally Sweden.

Gender gap ranking represents the position of a country in terms of gender equality (where one represents the best country in terms of gender equality). Between the considered countries, Sweden has the best gender gap ranking, followed by Italy and then India.

Unemployment rate before the first wave pandemic period in Sweden was 8.6%, in Italy 9.6% and in India 7%.

The average household size in Sweden is 2.2, in Italy 2.4 and in India is significantly higher at 4.8.

The political system in Sweden is a parliamentary representative democratic constitutional monarchy, Italy is a parliamentary republic and India is a federal parliamentary constitutional republic.

**Table:**

	Italy	Sweden	India
Density	200 hab/km^2	23 hab/km^2	411 hab/km^2
Population	60,244,639	10,327,589	1,352,617,328
Gender Gap Ranking	70°	3°	108°
Unemployment Rate	9.6%	8.6%	7%
Average Household Size	2.4	2.2	4.8

## Appendix B

### Restrictive measures in place when the data have been collected (20th April – 18th May 2020) in the major countries reached by our survey

#### Italy^3^

Italy started with first lockdown strategies from the 23rd February 2020 in some areas in the northern part of the country (Lombardy, Veneto and Emilia Romagna) and during March the restrictive measures have been expanded gradually. When the survey has been released, 20th April, the whole country was in lockdown:

- all schools and universities were closed,
- all shops were closed (except grocery stores, pharmacies, tobacconists, newsagents and petrol stations),
- all markets were closed,
- restaurants, pubs and cafes were closed (only home delivery allowed),
- all hair salons, beauticians and barbers were closed,
- banks, post and public offices were open,
- parks had been closed,
- only specific kinds of physical activities outside in constant motion were allowed (e.g. running, jogging),
- all the factories in non-essentials sectors were closed,
- it was forbidden to travel outside the municipality of residence (except for certified working reasons or serious health conditions).

The country started to open again from 4th May when it was allowed:

- to travel in the region of residence for certified reasons (e.g. work, health, visit relatives),
- to travel outside the region for certified reasons (e.g. work, health, urgent matters, coming back home),
- to access parks,
- to pick up take away food,
- to go back to work for workers in manufacturing and construction industry, as well as real estate agents and wholesalers,

while respecting social distancing and using facemasks in public places.

Since, there was progressive ease of the restrictions, in particular form 17th May most of the commercial activities were allowed to open and it was possible to travel freely within each region while respecting social distancing and using facemasks in public places.

Note that in Italy every region might have added more strict rules, but the baseline that everyone had to follow is described above. Our respondents are mainly from the northern part of Italy where more strict measures took place.

#### Sweden^4^

In Sweden, at the time of the survey, the following restrictions and guidelines were in place:

- all restaurants, bars, cafés, school dining halls and other venues serving food and beverages had to ensure that tables were spaced appropriately to avoid crowding and customers had to be always seated when consuming,
- it was prohibited to hold public gatherings and public events for more than 50 people,
- pharmacies were not allowed to dispense more medications than patients needed for a three-month period,
- it was not possible to visit the national care homes for the elderly,
- it was forbidden to leave home if experiencing any flu-like symptoms (e.g. coughing, cold, fever),
- it was recommended to work from home whenever possible,
- it was strongly recommended to keep a social distance of 2 m between people when possible.

Moreover, from April many companies started a voluntary lockdown of their facilities.

#### India^5^

India began the lockdown from 24th March extended to 31st May. From 20th April several relaxation measures started to support economic activities in regions not considered hotspots. From 29th April inter-state travel was allowed for working reasons. Further relaxations in many areas of the country for economic activities took place on 4th May.

#### France^6^

France started introducing restrictive measures from mid-March: school closure, ban of all non-essential activities, outings and long-distance travel. The lockdown started to ease from 11th May when primary schools, shops and industries started reopening, with some differences between regions.

#### Germany^7^

Since mid-March, Germany had in place several restrictive measures such as border closure, closure of schools and non-essential businesses, social distancing requirements and ban on public gathering. On 20th April smaller shops reopened while following social distancing. From 4th May some grades in schools started to reopen together with cultural and leisure venues. Soon after, the containment measures further eased for shops, restaurants and facilities for physical activities. From 16th May Germany started opening the borders to neighbouring countries.

## Appendix C

### Survey structure

**Table:**

*Before COVID-19*		
Question	**Activities**	**Options**
How often did you use to do these activities before the coronavirus outbreak?	Commute to work or school	Less than once a month
	Travel to grocery stores	Once a month
	Purchase groceries online	Once every second week
	Travel to non-grocery shopping	Once a week
	Purchase non-groceries online	2–4 times a week
	Order take away food	5–7 times a week
	Eat out in restaurants, bars, cafes	More than daily
	Travel to visit friends and family	
	Go out for entertainment/hobbies	
	Perform physical activities	
How did you use to travel to perform these activities before the coronavirus outbreak? If you use multimodal travelling, select the mode which covers the most distance and most commonly used.	Commute to work or school	Car (driver)
	Travel to grocery stores	Car (passenger)
	Travel to non-grocery shopping	Motorcycle
	Eat out in restaurants, bars, cafes	Public transport or train
	Travel to visit friends and family	Bicycle or by foot
	Go out for entertainment/hobbies	Other
	Perform physical activities	N/A
How long did it take to travel to these activities before the coronavirus outbreak? According to the mode selected above, estimate the average of one-way trip	Commute to work or school	< 10 min
	Travel to grocery stores	10-30 min
	Travel to non-grocery shopping	30-60 min
	Eat out in restaurants, bars, cafes	1–2 h
	Travel to visit friends and family	3–5 h
	Go out for entertainment/hobbies	> 5 h
	Perform physical activities	N/A
For how long did you use to use the internet connection for the following activities before the coronavirus outbreak? Answer considering an average in hours per week.	For entertainment	< 1 h
	For personal videocall/call/chat	1–2 h
	For work or study	3–5 h
	For work or study meetings and calls	6–10 h
		11–15 h
		15–20 h
		20–25 h
		> 25 h
To what extent does the coronavirus outbreak influence your daily life?		1 - not at all
		2
		3
		4 - a lot
*During COVID-19*		
Question	**Activities**	**Options**
How often do you do these activities now?	Commute to work or school	Less than once a month
	Travel to grocery stores	Once a month
	Purchase groceries online	Once every second week
	Travel to non-grocery shopping	Once a week
	Purchase non-groceries online	2–4 times a week
	Order take away food	5–7 times a week
	Eat out in restaurants, bars, cafes	More than daily
	Travel to visit friends and family	
	Go out for entertainment/hobbies	
	Perform physical activities	
How do you travel to perform these activities now? If you use multimodal travelling, select the mode which covers the most distance and most commonly used.	Commute to work or school	Car (driver)
	Travel to grocery stores	Car (passenger)
	Travel to non-grocery shopping	Motorcycle
	Eat out in restaurants, bars, cafes	Public transport or train
	Travel to visit friends and family	Bicycle or by foot
	Go out for entertainment/hobbies	Other
	Perform physical activities	N/A
How long does it take to travel to these activities now? According to the mode selected above, estimate the average of one-way trip	Commute to work or school	< 10 min
	Travel to grocery stores	10-30 min
	Travel to non-grocery shopping	30-60 min
	Eat out in restaurants, bars, cafes	1–2 h
	Travel to visit friends and family	3–5 h
	Go out for entertainment/hobbies	> 5 h
	Perform physical activities	N/A
For how long do you use the internet connection for the following activities now? Answer considering an average in hours per week.	For entertainment	< 1 h
	For personal videocall/call/chat	1–2 h
	For work or study	3–5 h
	For work or study meetings and calls	6–10 h
		11–15 h
		15–20 h
		20–25 h
		> 25 h
*Online shopping behaviours*		
Question	**Activities**	**Options**
Before the coronavirus outbreak, how much of your shopping was online?	Grocery shopping	0% [online]
	Other shopping	10%–30%
		40%–60%
		70%–90%
		100% [online]
How much of your shopping during the coronavirus outbreak is online?	Grocery shopping	0% [online]
	Other shopping	10%–30%
		40%–60%
		70%–90%
		100% [online]
Which kind of items do you usually shop during the coronavirus outbreak (not considering groceries)? [Multiple choice]		Clothes
		Hobbies related items (sport, art, music equipment, technology, stationery, books..)
		Items for the house, garden
		Work related items
		Others [Specify]
*Perceived safety and likelihood of keeping the new habits*		
Question	**Activities**	**Options**
During the coronavirus outbreak, how safe do you feel while engaging in the following activities, considering possible precautions that you take?	Travelling by public transport or train	1 - not safe at all
	Travelling by car	2
	Visiting stores	3
	Being at the workplace or school	4 - very safe
	Going to restaurants, pubs and cafes	
	Going to the gym	
	Spending time outside	
	Receiving home deliveries	
If you have changed your behaviour since the coronavirus outbreak, how likely are you to keep your new habits when the threat from the virus is removed?	Travel and commuting	1 - not likely
	Grocery shopping	2
	Shopping	3
	Work or study	4 - very likely
	Handle meetings at work or school	no change
	Free time	
	Physical activities	
*Demographics*		
Question	**Options**	**Number**
Where do you currently live?	List of UN recognized states	
What is your postal code?	Open question	
What is your country of origin?	List of UN recognized states	
What gender do you identify with?	Female	
	Male	
	Other [Specify]	
How many people in these age groups live in your household?	Children (< 18 years old)	None
	Adults	1–3
	Elderly (> 65 years old)	More than 3
What is your highest level of education?	No schooling completed	
	High school graduate, diploma or equivalent	
	Trade/technical/vocal training	
	Bachelor’s degree	
	Master’s degree	
	Professional degree	
	Doctorate degree	
	Other [Specify]	
What is your main employment?	Employee	
	Self-employed	
	Housewife/houseman	
	Student	
	Part time worker	
	Volunteering	
	Military	
	Retired	
	Unemployed	
	Other [Specify]	
Do you have any comment to add?	Open question	

## Footnotes

1 List of respondents per country: Italy (419); Sweden (226); India (69); France, Germany (8); Malaysia, United Kingdom (6); Netherlands, Spain (5); Belgium, Switzerland (4); Canada, Norway, Portugal (3); Denmark, Pakistan, United States (2); Algeria, Austria, Ireland, Latvia, Peru, Singapore (1).
